# Expanded Graphite-Based Materials for Supercapacitors: A Review

**DOI:** 10.3390/molecules27030716

**Published:** 2022-01-21

**Authors:** Dan Zhang, Chao Tan, Weizhuo Zhang, Weijie Pan, Qi Wang, Le Li

**Affiliations:** 1 Shaanxi Key Laboratory of Catalysis, School of Chemistry and Environment Science, Shaanxi University of Technology, Hanzhong 723001, China; zhangdan@snut.edu.cn (D.Z.); zd199725@163.com (C.T.); 2 Shaanxi Key Laboratory of Industrial Automation, School of Mechanical Engineering, Shaanxi University of Technology, Hanzhong 723001, China; zhangweizhuo@163.com (W.Z.); ppp18191091323@163.com (W.P.); wq19154052@163.com (Q.W.)

**Keywords:** expanded graphite, supercapacitor, symmetric supercapacitor, asymmetric supercapacitor, lithium-ion hybrid capacitors

## Abstract

Supercapacitors have gained e wide attention because of high power density, fast charging and discharging, as well as good cycle performance. Recently, expanded graphite (EG) has been widely investigated as an effective electrode material for supercapacitors owing to its excellent physical, chemical, electrical, and mechanical properties. Based on charge storage mechanism, supercapacitors have been divided into symmetric, asymmetric, and hybrid supercapacitors. Here, we review the study progress of EG-based materials to be electrode materials. Furthermore, we discuss the application prospects and challenges of EG-based materials in supercapacitors.

## 1. Introduction

**Figure 1:**
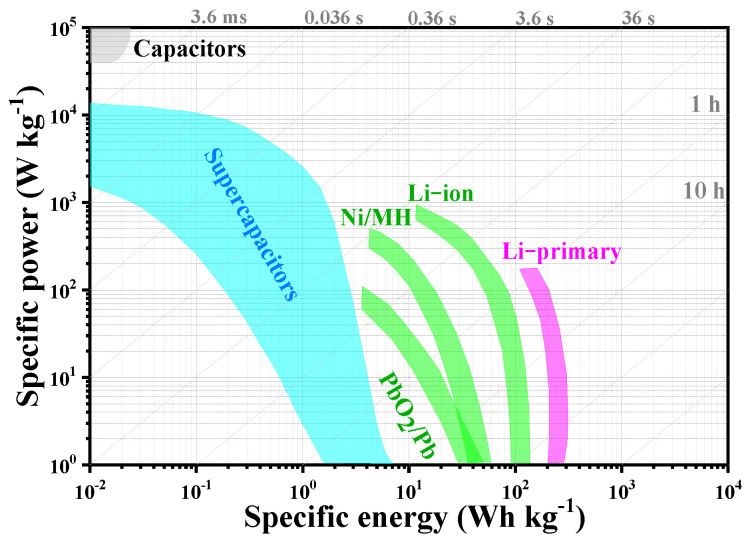
Ragone plot comparing the electrochemical properties of Li-primary battery, Li-ion battery, Ni/MH battery, PbO_2_/Pb battery, Supercapacitors [19]. Reprinted with permission from Reference [19]. Copyright 2021 Elsevier.

Owing to the reduction of fossil fuel reserves and global warming, developing alternative clean energy sources, including solar, wind, as well as tidal energy is urgent [1,2,3]. However, they are intermittent energy sources affected by the natural environment; thus, they need to be converted and stored, which has drawn researchers’ interest in the R&D of sustainable energy conversion and storage technologies [2,4,5,6]. Among a variety of energy storage devices, rechargeable batteries and supercapacitors have been the two dominating electrochemical energy storage technologies (Figure 1) [2,3]. Rechargeable batteries have been promising within consumer electronics together with electric vehicles because of their high energy density. However, their power density is limited due to the slow ion insertion/de-intercalation process in the electrode material [3,7,8,9,10]. When compared to rechargeable batteries, supercapacitors exhibit quicker charging and discharging (supercapacitors: 1–10 s vs. battery: 0.5–5 h), higher power density (supercapacitors: 500–10,000 W kg^−1^ vs. battery < 1000 W kg^−1^), remarkable longer life (supercapacitors > 500,000 h vs. battery: 500–1000 h), together with safer operation [2,11,12,13]. However, the low energy density of supercapacitors (supercapacitors: 1–10 W h kg^−1^ vs. battery: 10–100 W h kg^−1^) is a major challenge to the further development of supercapacitors [2,11,14,15,16,17,18]. To overcome this, most studies have focused on developing high-performance supercapacitor electrode materials.

**Figure 2:**
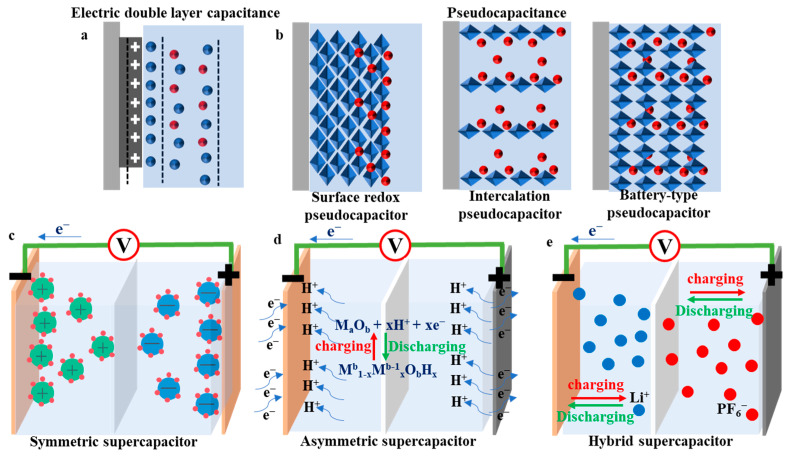
Schematic illustration of (**a**) EDLCs; (**b**) pseudocapacitance type of supercapacitor energy storage mechanisms; (**c**) symmetric supercapacitors; (**d**) asymmetric supercapacitors, (**e**) hybrid supercapacitors type of supercapacitor.

Generally, supercapacitors are categorized into two groups by charge storage mechanism: electric double layer capacitors (EDLCs) and pseudocapacitors (Figure 2a,b). Charge storage in EDLCs is achieved through charge separation within electrode/electrolyte interface [5,20,21]. This process only involves physical adsorption but not chemical reactions. Pseudocapacitors use fast, surface- and nondiffusion-limited redox reactions to store charges, resulting in pseudocapacitance or redox capacitance mechanism [4,5,22]. Considering the electrode composition, supercapacitors can be classed into symmetrical, asymmetrical, and hybrid supercapacitors (Figure 2c). Symmetrical supercapacitors combine the same material with the same capacitance on the anode and cathode or a device with a working electrode of an electric double layer or pseudocapacitance level based on the working mechanism of the electrode material [23]. Asymmetric supercapacitors combine two electrode materials with a good potential window [23,24]. As a new type of supercapacitor, hybrid supercapacitors are composed of battery-type negatives (electrochemical insertion or conversion) and capacitive positives (physical adsorption) and have many characteristics of supercapacitors [14,15,16,17,18].

Electrode material is vital in supercapacitors because it determines the capacitance, cycle, and rate performances of the supercapacitor [3]. Expanded graphite (EG) is obtained from expanded/split expandable graphite, which is the best prospective carbon anode material for different energy storage devices in recent years [25,26,27,28,29]. Noticeably, EG owns the uniform long-range-ordered layered structure, an enlarged interlayer distance, and honeycomb-like microstructures composed of plentiful translucent/wrinkled lamelliform graphene layers/sheets and turbostratic well-aligned structures, which provide a fantastically large surface area for admirable charge transfer [26,28]. Moreover, when compounded with other electrochemical activity materials, EG cannot provide a facile charge transfer network but cushion volume expansion and even provide additional pseudocapacitance [30,31]. EG-based materials show tremendous potential as high-performance electrode materials for supercapacitors because of the above-mentioned characteristics. However, no comprehensive reviews on the synthesis methods, composite strategies, and specific roles of EG-materials in supercapacitors are available yet. Here, first, we briefly introduce the definition and general synthesis method of EG. Then, we summarize recently developed strategies for the recombination of supercapacitors, and discuss the main role of EG-based materials in supercapacitors. Finally, we summarize the challenges and opportunities for developing EG and future research on supercapacitors.

## 2. Introduction and Synthesis of EG

**Figure 3:**
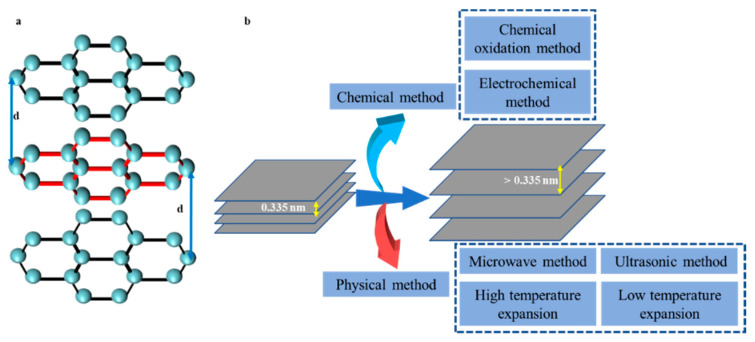
A brief diagram of structures of EG (**a**) and preparation of EG (**b**) [40]. Reprinted with permission from Reference [40]. Copyright 2021 RSC.

EG uses graphite as a raw material and intercalates suitable chemical substances between graphite layers in different ways to obtain expandable graphite (Figure 3a). In 1841, Schafhautl analyzed graphite wafers in a sulfuric acid solution and reported EG for the first time [32,33]. Since then, researchers have explored the synthesis and application of EG. Currently, the technology for preparing EG can be roughly classified into two categories: chemical and physical methods (Figure 3b). Chemical methods mainly include chemical oxidation [34] and electrochemical methods [35]. Among them, the chemical oxidation method is more commonly used, but this method uses strong oxidants in the preparation and generates environmental pollutants, which limits its use. The electrochemical method has attracted wide attention because it is a fast, efficient, and green method. However, this method uses graphite foil or graphite paper as an electrode and requires additional steps for further processing of the raw material graphite powder. High/low-temperature expansion [36,37], microwave [38], and ultrasonic methods [39] are other physical processes. The high/low-temperature expansion method is currently most commonly used, but it has some disadvantages such as excessive energy consumption, inert gas protection, and high equipment requirements. The microwave method for preparing EG is fast and efficient, but it consumes a lot of energy, and achieving further scale-up production is difficult, which limits its industrial application. The ultrasonic preparation of EG requires the selection of specific solvents and has low yields.

EG can be prepared in several ways. Using different methods results in differences in the structure and surface chemical state of EG, which greatly affect its electrochemical behavior and performance. Therefore, choosing a suitable EG synthesis method according to the purpose is vital in developing supercapacitor materials.

## 3. Application EG-Based Materials in Supercapacitor

EG has a long-range ordered layered structure, adjustable interlayer distance, abundant honeycomb microstructures, and turbine-layered ordered structures [25,26]. These features provide EG with a great surface area for excellent charge transfer and accelerate the kinetics of electrochemical de-intercalation of charges on graphite materials, thereby resulting in a high reversible capacity and high rate-ability [25,26,27,28,29]. In this section, we present the latest research results of EG-based materials in supercapacitors, including symmetrical, asymmetrical, and lithium-ion (Li-ion) hybrid supercapacitors.

### 3.1. Symmetric Supercapacitor

Symmetrical supercapacitors combine the same material with the same capacitance on the anode and cathode or a device with a working electrode of an electric double layer or pseudocapacitance level based on the working mechanism of the electrode material [23]. Symmetrical supercapacitors usually consist of two electrodes of the similar supercapacitor type, including carbon and pseudocapacitive materials. Most commercial supercapacitors are based on two symmetrical carbon electrodes in an organic electrolyte, and their voltage window can reach 2.7 V [5,39,40,41,42,43,44,45,46]. Additionally, the working voltage limit of the electrolyte solution is approximately 1.23 V, which is limited by water decomposition [5]. Consequently, widening the voltage window is key to further improving the energy density of water-electrolyte-based supercapacitors. The excellent interlayer spacing of EG-based materials facilitates the adsorption of ions in the electrolytes, which is expected to solve this problem.

**Figure 4:**
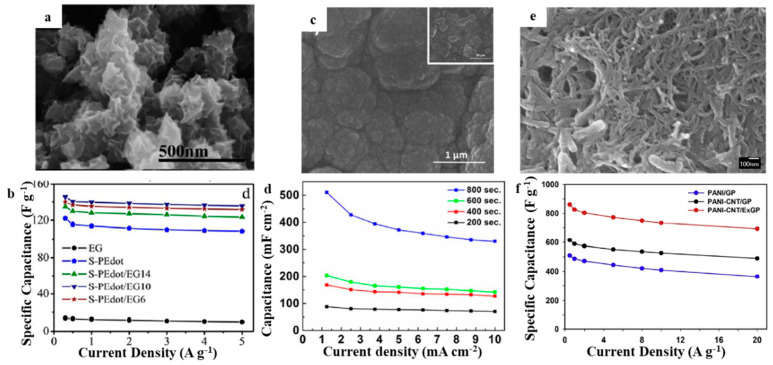
SEM of (**a**) S-PEdot/EG [47]; (**c**) EG–PPy [48]; (**e**) PANI-CNT/EG [50]; the specific capacitance of (**b**) S-PEdot/EG [47]; (**d**) EG–PPy [48]; (**f**) PANI-CNT/EG with various current densities [50]. Reprinted with permission from Reference [47]. Copyright 2016 Weily. Reprinted with permission from Reference [48]. Copyright 2018 IOPscience. Reprinted with permission from Reference [50]. Copyright 2018 Springerlink.

The interface interaction between EG and a conductive polymer can accelerate the transmission of electrolyte ions and electrons during charging/discharging, thereby significantly improving the electrochemical performance of hybrid materials. In 2016, Kang et al. prepared a nanohybrid of sulfamic acid-doped poly(3,4-ethylenedioxythiophene) produced upon EG (S-PEDOT/EG) with 3D hierarchical nanostructures through a surfactant-free in situ chemical oxidation polymerization [47]. As-prepared S-PEDOT/EG10 composite to be supercapacitor electrode material exhibited specific capacitance with 139.6 F g^−1^ under 1.0 A g^−1^. A two-electrode symmetric supercapacitor showed the high energy density of 6.83 W h kg^−1^ under the power density of 146 W kg^−1^ and remained 76.3% after 2000 cycles with 1.0 A g^−1^ (Figure 4a,b). These good characteristics result from 3D hierarchical structures of S-PEDOT/EG, EG and PEDOT molecule π–π interactions, together with sulfamic acid doping as a fixed counterion. Yuksel et al. prepared EG–polypyrrole hybrid (EG–PPy) nanocomposites by electrodeposition of PPy with a brush-coated EG electrode [48]. EG was used as a conductive layer for PPy deposition and charge collection in the nanocomposite. The as-prepared EG–PPy electrodes exhibited high specific capacitance (177.8 F g^−1^) and remarkable cycling stability (90.6% after 5000 cycles at 5.0 mA cm^−2^) (Figure 4c,d). Zhou et al. used surface treatment to prepare surface EG foils as substrates for electrochemical growth of polyaniline to prepare EG/polyaniline (EG/PANI) composites [49]. Because the EG/PANI composites exhibited excellent electron transmission paths at the interface of substrate and electrode material, the shrinkage together with expansion resistance were reduced. The resulting EG/PANI composite exhibited a high specific capacitance (422.1 mF cm^−2^ with 0.5 mA cm^−2^), excellent rate capability, together with good cycling life (94.1% of capacitance retention within 5000 cycles). Zhou et al. forwarded an easy electrochemical method to prepare EG and prepared a polyaniline/carbon nanotube composite electrode with EG as a substrate (PANI-CNT/EG) through the one-step co-electrodeposition method [50]. In the composite electrode, EG could offer plenty of contact points within the electrode material/current-collector interface, hence significantly decreasing shrinkage/diffusion resistance. The as-prepared PANI-CNT/EG composite exhibited the high specific capacitance of 826.7 F g^−1^. The as-assembled flexible solid-state supercapacitor, on the basis of PANI-CNT/EG composite, showed good flexibility and satisfying rate performance, high energy/power performance (7.1 kW kg^−1^ with the energy density of 12.0 W h kg^−1^), together with excellent cycling retention (77.6% capacitance retention for 3000 cycles) (Figure 4e,f).

**Figure 5:**
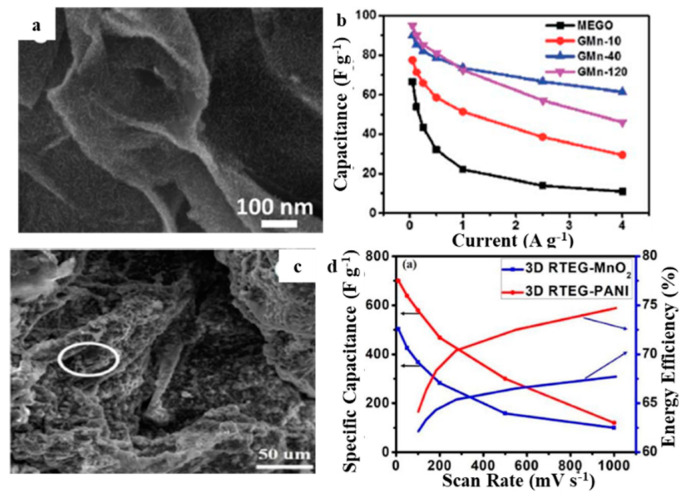
SEM of (**a**) MEGO–MnO_2_ [51], (**c**) 3D RTEG–MnO_2_ [52], the specific capacitance of (**b**) MEGO–MnO_2_ [51], (**d**) 3D RTEG–MnO_2_ and RTEG–PANI with various current densities [52]. Reprinted with permission from Reference [51]. Copyright 2016 RSC. Reprinted with permission from Reference [52]. Copyright 2019 IOPscience.

Similarly, forming a composite of metal oxide and EG can also promote EG electrochemical performance. Microwave-expanded graphite oxide (MEGO)–manganese dioxide (MnO_2_) hybrids with a 3D structure were prepared via the self-limiting redox reaction of MEGO and potassium permanganate [51]. The composite was composed of ultrathin MnO_2_ nanosheets attached to MEGO surface. Because of the short ion diffusion path, the conductivity was increased, improving the utilization efficiency of MnO_2_. The energy density of the symmetrical supercapacitor, based on the MEGO-MnO_2_ mixed material containing 24.5 wt% MnO_2,_ was 14 W h kg^−1^ (13.6 W h L^−1^), and the power density was 250 W kg^−1^ (243 W L^−1^), when the energy density was 5.46 W h kg^−1^ (5.3 W h L^−1^), the power density was 7.67 kW kg^−1^ (7.44 kW L^−1^) at a voltage of 2 V within 1 M sodium sulfate electrolyte (Figure 5a,b). Xiong et al. obtained a 3D ribbon-shaped thermally EG (3D RTEG)-based (MnO_2_ and PANI) composite material for supercapacitor electrodes by one-step electrochemical plug-electrode deposition of MnO_2_ or PANI into 3D RTEG [52]. Large specific capacitances of 500 F g^−1^ (~4 F cm^−2^) and 700 F g^−1^ (~6 F cm^−2^) were achieved for 3D RTEG–MnO_2_ and RTEG–PANI, respectively. Furthermore, both materials showed good energy efficiencies of 65–70% and 75–78%, respectively. Additionally, constructed supercapacitors with the 3D RTEG–MnO_2_ and RTEG–PANI hybrids exhibited good energy densities of 50.12 W h kg^−1^ (451.08 μW h cm^−2^) and 61.23 W h kg^−1^ (551.07 μW h cm^−2^) while retaining satisfying power densities of 15.26 W h kg^−1^ (137.34 mW cm^−2^) together with 20.15 kW kg^−1^ (181.35 mW cm^−2^), respectively (Figure 5c,d).

Metal sulfide/EG composites can also enhance EG electrochemical performance. Wei et al. successfully prepared the Ni–Co–S/EG composite electrode with a 3D nanosheet structure through the facile and green electrodeposition method [53]. Ni–Co–S/EG composite showed the high specific capacitance of 1516.5 F g^−1^ at 1 A g^−1^, a satisfying rate capability of 74.2% at 20 A g^−1^, together with a good capacity retention (84.4% capacity retention within 1000 cycles under 10 A g^−1^) due to the synergistic effects between Ni–S and Co–S.

**Figure 6:**
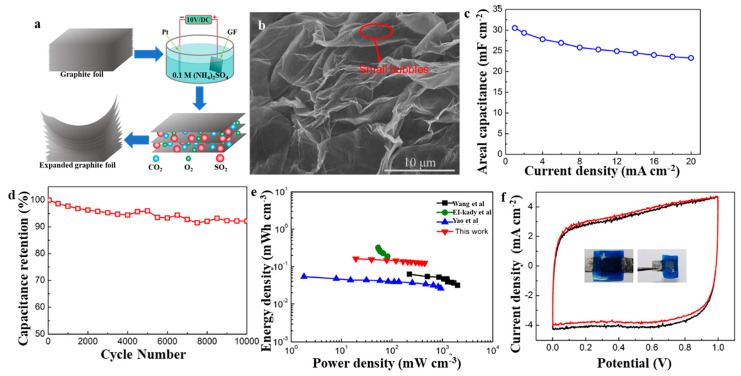
(**a**) Schematic illustration of the fabrication process of EG foil; (**b**) SEM of EG foil; (**c**–**f**) Electrochemical properties of solid flexible supercapacitors based on EG foil [35]. Reprinted with permission from Reference [35]. Copyright 2017 Elsevier.

EG has also been used to construct solid flexible supercapacitors. Li et al. showed the green and simple one-step method to prepare EG foil through electrochemical oxidation of EG foil in a salt solution [35]. An EG foil electrode with a distinct structure and good conductivity exhibited good supercapacitor performance (65 mF cm^−^^2^), satisfying rate capability (maintained 80% with the current density of 20 mA cm^−^^2^), and excellent capacity retention (95% capacitance remained after 10,000 cycles with 20 mA cm^−^^2^). Further, the flexible symmetric supercapacitor was fabricated with two electrodes and a sulfuric acid–polyvinyl alcohol gel to be a solid-state electrolyte. The symmetric supercapacitor delivered excellent areal capacitance (30.5 mF cm^−^^2^), high-rate performance (76% capacitance retained when the current density was increased from 1 to 20 mA cm^−^^2^). Additionally, the device also exhibited good capacity retention (92% capacitance after 10,000 cycles at current density of 20 mA cm^−^^2^). Moreover, the device characteristics didn’t exhibit any apparent fluctuate when it was bent 180°, indicating good mechanical flexibility (Figure 6). The EG-based symmetric supercapacitor as a traditional electrochemical energy storage device suffers from poor performance. Therefore, there are fewer studies in recent years.

**Table 1:** Summary of symmetric supercapacitor using EG-based materials as electrode materials.

Electrode Material	Voltage Window (V)	Electrolyte	Cycle Performance	Specific Capacitance	Energy Density (W h kg^−1^)	Power Density (W kg^−1^)	Ref.
SPEDOT/EG	−0.2–0.8	1 M LiClO_4_	76.3%, 2000 cycles, 1 A g^−1^	139.6 F g^−1^	6.83	146	[47]
MEGO–MnO_2_	0–2	1 M Na_2_SO_4_	-	97 F g^−1^	14	250	[51]
EG foil	0−1	0.1 M PVA + 0.1 M H_2_SO_4_	95%, 1000 cycles, 20 mA cm^−2^	65 F cm^−2^	19 mW h cm^−3^	447 mW cm^−3^	[35]
EG-PPy	0–0.8	PVA + H_2_SO_4_	90.6%, 5000 cycles, 0.5 mA cm^−2^	177.8 F g^−1^	-	-	[48]
PANI-CNT/ExGP	0–0.8	PVA + H_2_SO_4_	77.6%, 3000 cycles, 50 mV s^−1^	826.7 F g^−1^	7.1	12	[49]
EG/PANI	−0.5–0.5	1 M HCl	94.1%, 5000 cycles, 80 mV s^−1^	422.1 mF cm^−2^	58.4 μW h cm^−2^	9.4 mW cm^−2^	[50]
EG/CuO@C	−0.5–0.5	6 M KOH	87%, 8000 cycles, 10 mV s^−1^	335 F g^−1^	14.3	10.1	[54]
3D RTEG/MnO_2_	0–1.0	1.5 M Li_2_SO_4_	90%, 5000 cycles, 100 mV s^−1^	500 F g^−1^	50.12	15,260	[52]
3D RTEG/PANI	0–0.1	1.5 M Li_2_SO_4_	90%, 5000 cycles, 100 mV s^−1^	700 F g^−1^	61.23	20,150	[52]
Ni-Co-S/EG	0.15–0.55	6 M KOH	84.4%, 1000 cycles, 10 A g^−1^	1516.5 F g^−1^	-	-	[53]

Acronym definitions: Sulfamic acid-doped poly(3,4-ethylenedioxythiophene) grown on expanded graphite nanohybrids (SPEDOT/EG); Three-dimensional (3D) MnO_2_ structures on microwave-expanded graphite oxide (MEGO–MnO_2_); Expanded graphite-polypyrrole (EG-PPy); Polyaniline-carbon nanotube one-step co-electrodeposition expanded graphite composite (PANI-CNT/ExGP); Expanded graphite embedded with CuO nanospheres coated with carbon (EG/CuO@C); Three-dimensional ribboned thermally expanded graphite-based MnO_2_ bifunctional hybrid (3D RTEG/MnO_2_); Three-dimensional ribboned thermally expanded graphite-based PANI bifunctional hybrid (3D RTEG/PANI).

### 3.2. Asymmetric Supercapacitor

Asymmetric supercapacitors can be defined as a combination of two electrode materials with a good potential window [23,24]. An asymmetric supercapacitor consists of two different supercapacitor-type electrodes; one is the double-layer carbon materials, while the other electrode is the pseudocapacitance material. During the charging and discharging process, the asymmetric supercapacitor can fully utilize various potential windows in two electrodes for maximizing the working voltage of the entire device [24,55,56,57,58,59,60,61].

**Figure 7:**
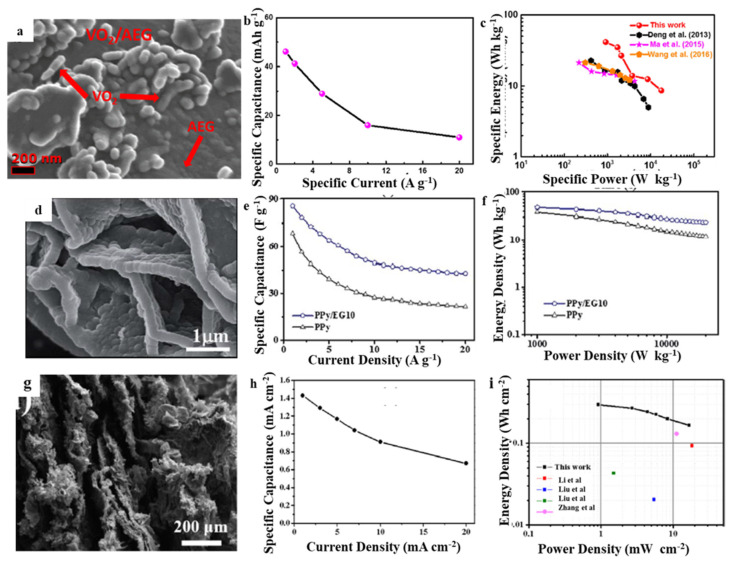
SEM of (**a**) VO_2_/AEG [64], (**d**) PPy/EG [65]; (**g**) EGP [31]; the specific capacitance of (**b**) VO_2_/AEG [64]; (**e**) PPy/EG [65]; (**h**) EGP [31] with various current densities; Ragone plots of volume power density versus energy density for (**c**) VO_2_/AEG [64]; (**f**) PPy/EG [65]; (**i**) EGP [31]. Reprinted with permission from Reference [64]. Copyright 2019 Elsevier. Reprinted with permission from Reference [65]. Copyright 2019 RSC. Reprinted with permission from Reference [31]. Copyright 2019 Elsevier.

Because the rich interlayer structure of EG is easy to compound with metal oxides, hydroxides and conductive polymers, EG-based composites are often used as cathode materials for asymmetric supercapacitors. In 2017, Barzegar et al. used EG and pine cone biomass as raw materials to prepare activated EG-composite by activating in potassium hydroxide [30]. Electrochemical performance of novel materials within the two-electrode configuration to be supercapacitor electrode showed the specific capacitance of 69 F g^−1^ under 0.5 A g^−1^ and a satisfying energy density of 24.6 W h kg^−1^ with the power density of 400 W kg^−1^. Yuan et al. prepared EG as a raw material and a layered Ni(OH)_2_/EG hybrid within an N, N-dimethylformamide-water system through in-situ electrodeposition [62]. The composite electrode showed the excellent initial specific capacitance of 1719.5 F g^−1^ under 1 A g^−1^ and good rate performance (1181.3 F g^−1^ under 10 A g^−1^) resulting from high capacitive characteristics chiefly derived from the synergistic impact in the layered Ni(OH)_2_/EG composite. Moreover, the constructed active carbon (AC)//Ni(OH)_2_/EG device exhibited a high energy density (32.3 W h kg^−1^ at a power density of 504.7 W kg^−1^) togrther with a long cyclic stability (retaining 79% capacitance within 1000 cycles of 5 A g^−1^). Similarly, Ni(OH)_2_/EG hybrid was prepared by the green microwave-assisted approach, while Ni(OH)_2_ particles were evenly distributed on the EG layers’ surface, helped to obtain the high specific capacitance with 1569 F g^−1^ under 1 A g^−1^ [63]. Additionally, the as-assembled AC//Ni(OH)_2_/EG asymmetric supercapacitors exhibited an energy density of 37.7 W h kg^−1^ at a power density of 490.9 W kg^−1^, 26.1 W h kg^−1^ even at a high-power density of 10.1 kW kg^−1^. Furthermore, when the current is 0.5 A g^−1^ and the voltage window is 1.6 V, it can get the maximum specific capacitance with 86.4 F g^−1^. When the current density is 5 A g^−1^ after 1000 cycles, specific capacitance remains at 80.1%. Ndiaye et al. synthesized vanadium dioxide/activated EG (VO_2_/AEG) hybrid material and carbon–vanadium–oxynitride (C–V_2_NO) porous network structure using chemical vapor deposition [64]. The electrochemical characteristics of the hybrid material (VO_2_/AEG//C–V_2_NO) was measured in a two-electrode asymmetric device by VO_2_/AEG composite to be anode and C–V_2_NO to be cathode with a 6 M KOH electrolyte. The asymmetric device delivered a specific energy of 41.6 W h kg^−1^ with a specific power of 904 W kg^−1^ under 1 A g^−1^ specific current and a high operating voltage of 1.8 V. Specific energy of 9 W h kg^−1^ was retained under an amplified specific current of 20 A g^−1^ with the specific power of 18 kW kg^−1^. The supercapacitor showed a 93% capacity retention after 10,000 constant gravimetric current cycles life with the specific current of 10 A g^−1^ and an excellent rate capability. It maintained significant device stability without any failure after voltage-floating tests in 100 h or above (Figure 7a–c). Wang et al. prepared PPy/EG nanohybrid by vacuum-assisted intercalation in-situ oxidative polymerization [65]. The as-prepared PPy/EG10 sample having 10% EG content exhibited excellent specific capacitance of 454.3 and 442.7 F g^−1^ at 1.0 A g^−1^, and specific capacitance cyclic stability rates of 75.9% and 73.3% at 15.0 A g^−1^ within 1 M H_2_SO_4_ as well as 1 M KCl electrolytes, respectively. The two-electrode symmetric supercapacitor exhibited an excellent energy density of 47.5 W h kg^−1^ at 1 kW kg^−1^ and could keep high cycle stability after 2000 cycles (Figure 7d–f). The 3D structure of the PPy/EG nanohybrid filters electrolytes and diffusion of ions, which improves the pseudocapacitance of polypyrrole. During charging and discharging, EG nanosheets act as collectors, accelerating electrons transfer. EG within the nanohybrid acts to be a self-supporting skeleton. It prevents volume expansion and contraction of the nanohybrid as well as improving the nanohybrid cycle stability. Murovhi et al. successfully synthesized α-manganese dioxide/activated EG (α-MnO_2_/AEG) composites with the easy hydrothermal method [66]. Under 1 A g^−1^, its maximum specific capacitance of three-electrode test has been 185.5 F g^−1^. Under 5 A g^−1^, the half-cell obtained 99.7% efficiency in 2000 cycles. The assembled device with the α-MnO_2_/AEG hybrid and AC-PVA composite as anode and cathode, respectively, showed good capacitive properties with a specific energy of 33 W h kg^−1^ under specific power of 999 W kg^−1^ at 1 A g^−1^ within 2.0 V cell potential. It showed with the 5 A g^−1^ specific current, after more than 10,000 cycles, 97.8% of high cycle life was obtained. This device cycle stability has been further evaluated through carrying out the voltage keeping for more than 70 h and remained 70% of initial capacitance with 5 A g^−1^ was maintained. Wang et al. prepared partially exfoliated graphite paper (EGP) through the cathode electrochemical method of tetrabutylammonium cation intercalation [31]. The prepared EGP exhibited a large specific surface area together with excellent electronic conductivity, which was an ideal substrate to in situ growth of NiCo–CH nanowires encapsulating graphene nanosheets by a simple hydrothermal approach. Because of the promoted electrolyte ion transfer and fast electron transmit, NiCo–CH@EGP achieved a good areal capacity of 2.55 C cm^−2^ with 0.5 A cm^−2^ as well as maintained 1.38 C cm^−2^ even at 60 mA cm^−2^. The constructed NiCo–CH@EGP//AC asymmetric supercapacitor exhibited an excellent energy density of 0.30 mW h cm^−2^ with a power density of 0.92 mW cm^−2^ together with a satisfying cycle life, retaining 78.1 % after 10,000 cycles at 20 mA cm^−2^ (Figure 7g–i).

**Figure 8:**
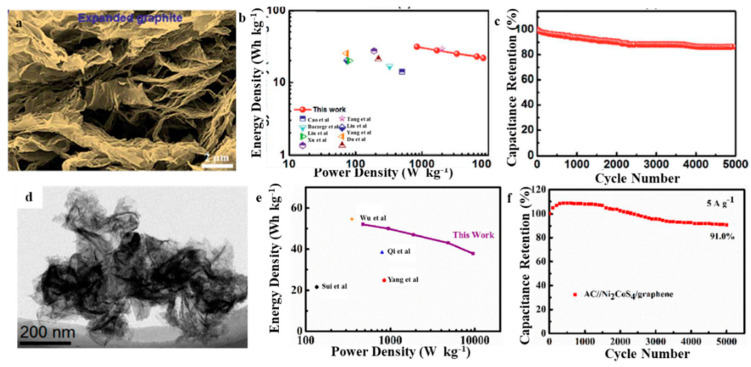
(**a**) SEM of EG [67]; (**d**) TEM of Ni_2_CoS_4_/EG [68]; Ragone plots of volume power density versus energy density for (**b**) EG [67]; (**e**) Ni_2_CoS_4_/EG [68]; Cycling performance of (**c**) EG [67]; (**f**) Ni_2_CoS_4_/EG [68]. Reprinted with permission from Reference [67]. Copyright 2018 RSC. Reprinted with permission from Reference [68]. Copyright 2019 Springerlink.

Similarly, EG-based composite materials are often employed as negative electrode materials to asymmetric supercapacitors. Chen et al. inserted alkyl amines into layered molybdenum trioxide and then carbonized them in situ at high temperatures to prepare MoO_3_/C nanocomposites [67]. The prepared MoO_3_/C as a supercapacitor electrode delivered an excellent specific capacitance (335 F g^−1^ at 1 A g^−1^) and a high rate of characteristics (70% capacitance retention rate from 1 to 10 A g^−1^). The as-assembled MoO_3_/C//EG asymmetric supercapacitor delivered a specific capacitance (88 F g^−1^ at 1 A g^−1^) and a satisfying specific energy density (31.3 W h kg^−1^ with a power density of 838.4 W kg^−1^) in 0–1.6 V voltage range and excellent capacity retention properties (86.5% capacity retention after 5000 cycles with 1 A g^−1^) (Figure 8a–c). The excellent performance is attributed to (1) opening of the MoO_3_ layer with conductive carbon, which can provide more redox sites for the Faraday reaction and promote the transfer of electrons and (2) the formation of a sandwich-type hybrid nanostructure by molybdenum trioxide and the embedded carbon layer, which facilitates the penetration and diffusion of electrolyte ions. Using a fast and energy-saving microwave heating approach, Ni_2_CoS_4_/EG hybrids were prepared within a mixed solvent of ethylene glycol and water [68]. The specific capacitance of the Ni_2_CoS_4_/EG hybrids could reach up to 2056.8 F g^−1^ under 5 A g^−1^, and specific capacitance would be 1923.3 F g^−1^ even under the 30 A g^−1^ current density; therefore, 92.5% of rate performance was obtained with the increase of current density from 5 to 30 A g^−1^. The composite also exhibited good stability of 94.4% when cycling with a current density of 30 A g^−1^ for 2000 cycles. It showed good initial capacitance, high-rate performance, together with excellent cycle life. Moreover, the constructed AC//Ni_2_CoS_4_/EG asymmetric supercapacitor exhibited an excellent specific capacitance of 120.3 F g^−1^ under 0.5 A g^−1^, a good cycle stability (91% under 5 A g^−1^ for 5000 cycles), and a high energy density of 52 W h kg^−1^ under 477 W kg^−1^ (Figure 8d–f). The EG-based symmetric supercapacitor combines the advantages of electric double-layer capacitance and pseudocapacitor, and the tunable interlayer spacing of EG is easy to combine with other electrochemically excellent materials. Therefore, it has become a research hotspot in recent years.

**Table 2:** Summary of asymmetric supercapacitor using EG-based materials as electrode materials.

Devices (Positive//Negative)	Voltage Window (V)	Electrolyte	Cycle Performance	Specific Capacitance	Energy Density (W h kg^−1^)	Power Density (W kg^−1^)	Ref.
MEGO-MnO_2_//Activated MEGO	0–1.8	1 M Na_2_SO_4_	75%, 5000 cycles, 1 A g^−1^	56 F g^−1^	25.1	93	[30]
AEG//APC	0–1.6	PVA + KOH + carbon black	-	69 F g^−1^	24.6	400	[62]
Ni(OH)_2_/EG//AC	0.1−0.6	6 M KOH	79%, 1000 cycles, 5 A g^−1^	1719.5 F g^−1^	32.3	504.7	[62]
Ni(OH)_2_/EG//AC	0.15–0.55	6 M KOH	80.1%, 1000 cycles, 5 A g^−1^	86.4 F g^−1^	37.7	490.9	[63]
MoO_3_/C//EG	0–1.6	1 M H_2_SO_4_	86.5%, 5000 cycles, 1 A g^−1^	88 F g^−1^	31.3	838.4	[67]
AC//Ni_2_CoS_4_/EG	0–0.55	6 M KOH	91%, 5000 cycles, 5 A g^−1^	120.3 F g^−1^	52	477	[68]
VO_2_/AEG//C-V_2_NO	0–1.8	6 M KOH	93%, 10,000 cycles, 10 A g^−1^	47 F g^−1^	41.6	904	[64]
PPy/EG//Graphite	0–2.0	1 M H_2_SO_4_	86.1%, 2000 cycles, 10 A g^−1^	454.3 F g^−1^	47.5	1000	[65]
α-MnO_2_/AEG//AC-PVA	0–1.0	1 M Na_2_SO_4_	99.7%, 2000 cycles, 5 A g^−1^	185.5 F g^−1^	33	999	[66]
NiCo-CH@EGP//AC	0–0.6	2 M KOH	78.1%, 10,000 cycles, 20 mA cm^−2^	2.55 C cm^−2^	0.30 mW h cm^−2^	0.92 mW cm^−2^	[31]

Acronym definitions: Three-dimensional (3D) MnO_2_ structures on microwave-expanded graphite oxide (MEGO–MnO_2_); Activated pinecone carbon (APC); Activated expanded graphite (AEG); Activated carbon (AC); Vanadium dioxide/activated expanded graphite (VO_2_/AEG); Carbon-vanadium oxynitride (C-V_2_NO); Polypyrrole/expanded graphite (PPy/EG); Alpha-manganese dioxide/activated expanded graphite (α-MnO_2_/AEG); Activated carbon-polyvinyl alcohol (AC-PVA).

### 3.3. Li-Ion Hybrid Capacitor

Li-ion capacitors (LICs) are made up of a capacitor-type cathode, one battery-type anode, and one appropriate electrolyte [15,69,70,71,72,73,74]. They rely on the surface reaction of the cathode and the lithiation/electrolysis of the anode to achieve energy storage and conversion [14,16,17,18]. Owning to the higher power density together with the longer cycle life than those of Li-ion batteries, as well as higher energy density than that of supercapacitors, LICs are regarded to be one of the most prospective electrochemical energy storage devices. However, because of the dynamism balance between the two electrodes, the actual energy/power output of LICs is poor. Improving the high capacitance of the cathode material and increasing the rate capability of the anode are key ways of improving LIC’s good electrochemical characteristics.

**Figure 9:**
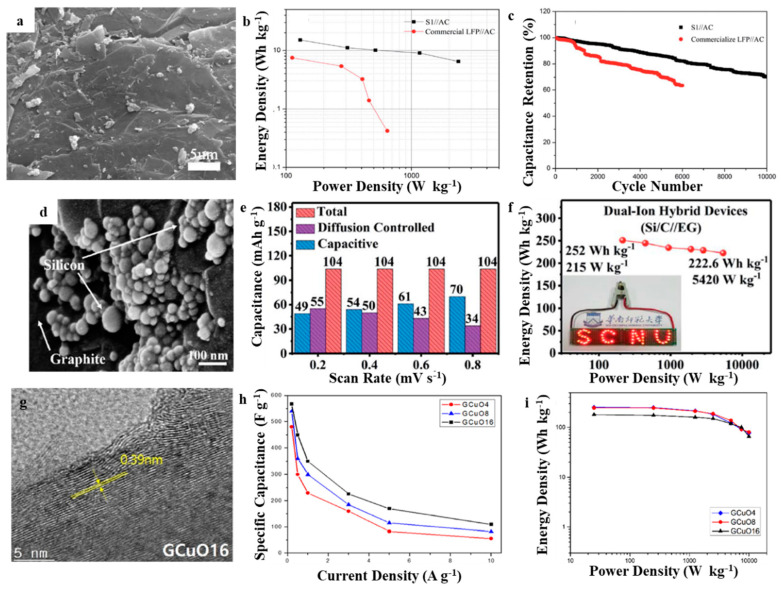
SEM of (**a**) LFP/EG [76]; (**d**) Si/C [78]; (**g**) GCuO [79]; Ragone plots of volume power density versus energy density for (**b**) LFP/EG [76]; (**f**) Si/C [78]; (**i**) GCuO [79]; the specific capacitance of (**c**) Si/C [78]; (**e**) GCuO [79]; (**h**) Si/C [78]. Reprinted with permission from Reference [76]. Copyright 2017 Elsevier. Reprinted with permission from Reference [78]. Copyright 2020 RSC. Reprinted with permission from Reference [79]. Copyright 2020 Elsevier.

Li et al. made lithium iron phosphate (LiFePO_4_)/EG (LFP/EG) composites through in situ sol–gel method [75]. The LFP/EG composite was used to be the anode, activated carbon to be the cathode, and a lithium nitrate aqueous solution to be the electrolyte for fabricating a LIC. The specific capacitance of optimized LFP/EG composite at 5 mV s^−1^ was 326.23 F g^−1^. The optimized LIC suggested a high specific capacitance at 200 mA g^−1^ was 53.31 F g^−1^. The LFP/EG composite and LIC maintained 84.8 % and 84.6 % of their initial specific capacitance when having 100 cycles, respectively. Qin et al. impregnated lithium dihydrogen phosphate and ferric citrate precursor solutions in a vacuum through an in situ sol–gel process and calcined them to form LFP/EG nanohybrids [76]. The hybrid material consists of spherical LFP particles embedded in the EG pores and wrapped with an EG film to form an efficient and stable conductive network. This form greatly accelerates the diffusion of Li-ions and improves their exchange between the LFP and the electrolyte. The LFP/EG nanohybrids showed a satisfying high-rate capability, good stability, and high specific capacitance of approximately 1200 F g^−1^. Furthermore, after 500 cycles, the LFP/EG hybrids-based LIC retained 100% of its initial capacitance (Figure 9a–c). Lv et al. prepared LFP/EG nanohybrids through a facile one-step method. They embedded spherical LFP nanoparticles with controllable size and good agglomeration in EG pores and wrapped them with an EG film [77]. This morphology formed an efficient and stable conductive network, promoting Li^+^ diffusion and exchange of LFP and the electrolyte. Thus, the LFP/EG composite exhibited good rate performance and cycle reversibility. LFP/EG//AC LICs were constructed in a LiNO_3_ electrolyte with the LFP/EG composite and AC as the anode and cathode, respectively. The as-assembled LIC showed a power density of 2367.9 W kg^−1^ with the energy density of 6.5 W h kg^−1^, excellent rate performance, and good cycling stability with 82.1% capacitance retention under 2 A g^−1^ within 6000 cycles. A dual-ion hybrid energy storage device with EG as the cathode and graphite/nanosilicon@carbon (Si/C) as the anode was fabricated for effective energy storage [78]. The Si/C//EG device showed a maximum specific capacitance of 185.5 F g^−1^, excellent cycling life of 94.4% when having 200 cycles with a current density of 30 A g^−1^, and energy densities of 252–222.6 W h kg^−1^ with power densities of 215–5240 W kg^−1^ (Figure 9d–f). Lee et al. fabricated high-energy-density hybrid LICs using graphite/copper oxide composite (GCuO) to be the negative electrode together with porous carbon (PC) to be the positive electrode [79]. The hybrid devices use the Faraday insertion/de-insertion and conversion reaction at GCuO and the adsorption/desorption of Faraday ions at PC. These LICs provided an excellent specific capacitance of 185.5 F g^−1^, a high specific energy density of 212.3 W h kg^−1^ with a specific power density of 1.3 kW kg^−1^ and maintained 85% of its initial energy density when having 500 cycles (Figure 9g–i). As an emerging electrochemical energy storage device, EG-based LICs have better specific capacitance, energy density, and power density than the aforementioned symmetric and asymmetric supercapacitors, however their energy storage mechanism is not fully understood and the manufacturing cost limits its further application. Therefore, this point should be paid attention to in the follow-up research.

**Table 3:** Summary of hybrid supercapacitor using EG-based materials as electrode materials.

Devices (Positive//Negative)	Voltage Window (V)	Electrolyte	Cycle Performance	Specific Capacitance	Energy Density (W h kg^−1^)	Power Density (W kg^−1^)	Ref.
LFP/EG//AC	0–1.0	1 M LiNO_3_	84.8%, 100 cycles, 5 mV s^−1^	326.23 F g^−1^	-	-	[75]
LFP/EG//AC	−0.6–1.0	1 M LiNO_3_	100%, 500 cycles, 1 A g^−1^	1200 F g^−1^	-	-	[76]
LFP/EG//AC	−0.6–1.0	1 M LiNO_3_	82.1%, 6000 cycles, 2 A g^−1^	44.7 F g^−1^	6.5	2367.9	[77]
EG//Si/C	3.0–5.0	4 M LiPF_6_	90%, 100 cycles, 0.1 A g^−1^	109.7 mAh g^−1^	252–222.6	215–5420	[78]
EG/CuO//porous carbon	1.0–4.0	1 MLiPF_6_	85%, 500 cycles, 0.1 A g^−1^	568.07 mAh g^−1^	212.3	1300	[79]

Acronym definitions: LiFePO_4_/expanded graphite (LFP/EG); Graphite@nano-silicon@carbon (Si/C); Expanded graphite/copper oxide composite (EG/CuO).

## 4. Summary and Outlook

Supercapacitors (electrochemical capacitors), drew extensive attention within various electrochemical energy storage devices for its outstanding power density, extraordinarily quick charging time, good low-temperature characteristics and long cycle life. EG has excellent electrical conductivity, distinct physical and chemical characteristics, and excellent electrical and mechanical properties; thus, it has broad application prospects in the field of supercapacitors. In the past seven years, the electrochemical performance and applications of EG as a supercapacitor electrode material have been rapidly developed.

This article reviews the research progress of EG-based materials as an electrode material (Table 1, Table 2 and Table 3) and their application prospects and challenges in supercapacitors. Although the application of EG-based materials in supercapacitors has made great progress, some challenges need to be surmounted.

1. Green, efficient, and controllable syntheses with interlayer spacing are prerequisites for obtaining advanced EG-based materials. However, the current preparation methods for EG are mainly restricted by the excessively long expansion time, severe environmental pollution, and the difficulty to control the size of the interlayer spacing, and these limit the application of EG. Developing a low-cost and green preparation process with a high yield and an adjustable number of layers has been a difficult problem in EG research.
2. Choosing other suitable materials to compound with EG is of great significance for constructing high-performance supercapacitor electrodes. EG exhibits excellent electrochemical performance, thus, is an excellent candidate material to practical supercapacitors. Nevertheless, its low energy and power densities do not conform with the requirements for practical applications. One of the shortcomings can be overcome by compounding other pseudocapacitive materials. Therefore, choosing the right composite material is crucial to improving the energy and power densities of EG-based supercapacitors.
3. To control the interface reaction between an EG-based electrode and electrolyte, thorough comprehension for the energy storage mechanism is necessary. There is also a need for further simulations and modeling to reveal potential electrochemical mechanisms in the nanoscale. Many theoretical and computational researches on EDLCs have been published. However, due to surface redox and ion intercalation pseudocapacitances have been more complex and arduous for simulation, the pseudocapacitances theoretical understanding is limited. What is more, the latest in situ microscopy and spectroscopy techniques provide direct experimental proof for them.
4. Apart from energy and power densities, the self-discharge and high and low temperature of EG-based supercapacitors should be noted. Supercapacitors have good high- and low-temperature performance, but they exhibit rapid self-discharge. Therefore, observing the self-discharge and high- and low-temperature performance of EG-based supercapacitors in future research is important. Furthermore, the reliability of devices based on EG-based supercapacitors such as high temperature storage, damp heat test, rapid temperature change, vibration, safety test (flammability, pressure relief, puncture, extrusion, impact, punching) should also be concern.
5. The rapid popularization of smart electronic products requires the continuous development of stimulus–response integrated smart power supplies. To gather more functions into an electrochemical energy storage device has been an interesting challenge. With more in-depth study, novel supercapacitors will take a vital role in providing lightweight, flexible, and wearable supercapacitors in the future.

## Data Availability

Not applicable.
